# *Chromobacterium violaceum* causing deadly sepsis

**DOI:** 10.18502/ijm.v12i4.3941

**Published:** 2020-08

**Authors:** Arun Sachu, Sunil Antony, Philip Mathew, Sanjo Sunny, Jency Koshy, Vijaya Kumar, Renu Mathew

**Affiliations:** 1 Department of Microbiology, Believers Church Medical College Hospital, Thiruvalla, India; 2 Department of Medicine, Believers Church Medical College Hospital, Thiruvalla, India; 3 Department of Critical Care, Believers Church Medical College Hospital, Thiruvalla, India

**Keywords:** *Chromobacterium violaceum*, Septicemia

## Abstract

Infections caused by *Chromobacterium violaceum* are extremely rare but can be relatively fatal in septicemia. We report a case of a 76 year old female who presented with pustules in the skin and later developed into septicemia. She succumbed to the illness despite escalating the antibiotic therapy to meropenem. To the best of our knowledge this is the 16^th^ case report from India.

## INTRODUCTION

*Chromobacterium violaceum* is a Gram negative bacillus that is not a part of normal human flora. It usually inhabits soil and stagnant water in tropical and subtropical regions (1). *Chromobacterium violaceum* produces a non diffusible pigment called violacein, which is soluble in ethanol and insoluble in water and choloroform (2). The first case of human infection by *Chromobacterium violaceum* was described in 1927 in Malaysia and since then about 200 cases have been described around the world (3). In India only a few cases have been reported with various clinical presentations at different locations (4, 5). Infection caused by *Chromobacterium violaceum* has an extremely high fatality rate especially when associated with septicemia. The mortality rate due to this organism ranges from 57% in the United States to 80% in India (6, 7). The objective of this case report is to generate knowledge regarding this rare but fatal organism and their antibiogram for successful management in the future.

## CASE REPORT

A 76 year old female who was a diagnosed case of *Myelodysplastic syndrome*, Autoimmune Hemolytic anaemia, Type 2 Diabetes Mellitus got admitted in our hospital. She presented with intermittent high grade fever. There was no history of altered sensorium, vomiting, chest pain, dysuria, altered bowel and bladder habits. This patient had numerous admissions in our hospital in the last 2 years due to recurrent episodes of urinary tract infection with different bacteria. She was also treated for typhoid fever 5 months back. Patient gave a history of washing her clothes in nearby lake suggesting a possible exposure to contaminated water.

### Clinical examination.

Patient was conscious and oriented. Vitals were stable. There were scattered and discrete papulo-pustular lesions over the body, the largest of which was on the right forearm measuring 5 × 6 cm. Other systemic examination was unremarkable. The treating team suspected a Gram positive skin and soft tissue infection and the patient was empirically started on amoxicillin-clavulanic acid and clindamycin.

Blood Investigations revealed anaemia, leukocytosis and thrombocytopenia.

Incision and drainage was done for the largest lesion on the right forearm and the pus collected was sent for culture. Blood was collected with complete aseptic precautions into aerobic blood culture bottles (Bact T/ ALERT/ 3D; Biomerieux, Marcy L Etoile, France).

Both Blood cultures became positive after 12 and 15 hrs of incubation respectively. Gram stain from the broth revealed Gram negative bacilli. Blood culture broth was inoculated into Blood and MacConkeys agar.

The condition of the patient deteriorated within 48 hrs and she developed hypotension. She was shifted to the intensive care unit, antibiotics escalated to meropenem and was initiated on ionotropic supports. There was a significant reduction in total leucocyte count and platelet count (Table 1). She developed acute kidney injury, her general condition continued to worsen, and succumbed to her illness the following day.

**Table 1.** Laboratory Investigations

**Lab parameters (Normal Range)**	**Day 1**	**Day 3**
Haemoglobin g/dl (11–15)	8.7	7.5
Total Leucocyte count/μL (4800–10000)	15480	8500
Platelet count Lakh/μL (1.5–4.5)	86000	42000
Serum Creatinine mg/dl (0.5–1.2)	1.79	2.98
Blood Urea mg/dl (17–49)	71.2	NA
C-Reactive protein mg/L (0–10)	190.9	NA

NA-Not Applicable.

**Fig. 1.**
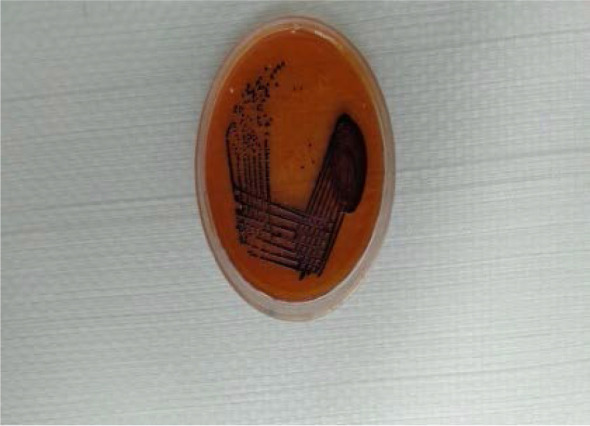
Pigmented colonies of *Chromobacterium violaceum*

After overnight incubation blood agar showed beta-haemolytic round colonies with blackish pigmentation. MacConkey agar showed non lactose fermenting colonies with blackish pigmentation (Fig. 1). Pus culture also showed similar colonies. Routine biochemical reaction was put up and further identification was done by VITEK-2 (Biomerieux, France). Organism was catalase and oxidase positive and indole negative. Urea was not hydrolysed, citrate was not utilized. Glucose was fermented with the production of acid. It was motile but did not ferment mannitol. Triple sugar iron revealed alkali/acid reaction without production of hydrogen sulphide.

**Fig. 2.**
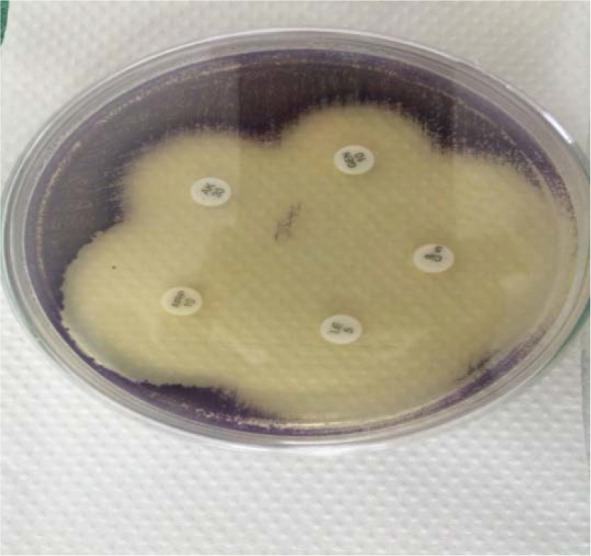
Antimicrobial susceptibility pattern of *Chromobacterium violaceum* on Muller Hinton agar

Organism was identified as *Chromobacterium violaceum* by VITEK-2 with 99% probability. It was found to be sensitive to amikacin, gentamicin, ciprofloxacin, levofloxacin and meropenem (Fig. 2.) but was resistant to ampicillin, amoxi-clav and cephalosporins. The results were interpreted according to Clinical and Laboratory Standards Institute (CLSI) guidelines for other non-Enterobacteriaceae. The isolation of the same organism from two blood cultures and pus revealed the pathogenic role of the organism.

## DISCUSSION

*Chromobacterium violaceum*, a tropical pathogen, was described for the first time in 1872 by Schroeter. It is motile, Gram negative, facultative anaerobic bacillus and took its name from the purple pigment – violacein (8). The fact that there are only few reports of human infections with this organism is a surprise, given the ease with which this organism is isolated from the soil and stagnant water bodies in the tropics and subtropics (9). Mostly human infection is seen in paediatric and young adults (3). Neonatal septicemia caused by *Chromobacterium violaceum* was described by Tiwari et al. (10).

The most common mode of transmission is the exposure of wounds and traumatic lesions to soil and stagnant water containing the organism (11, 12). Our case presented with pustules in the skin and then developed into septicemia. So the mode of transmission is probably due to exposure of the wound to soil and stagnant water. Infection can also occur due to ingestion of contaminated water but our patient had no history of diarrhoea and hence can be ruled out (13). Other unusual routes of exposure include scuba diving or near drowning and rarely following surgical procedures (7). Our case was a 76 year old woman, which to our knowledge is the oldest case of *Chromobacterium violaceum* causing sepsis.

*C. violaceum* septicemia is usually associated with chronic granulomatous disease (CGD), neutrophil dysfunction and G6PD deficiency (12). Our patient being immunosuppressive probably would have progressed to sepsis, eventually leading to her death. Diagnosis of *C. violaceum* requires high index of clinical suspicion and isolation of the organism from clinical specimen. A Multiplex PCR for detection of *C. violaceum* was described by Scholz et al. but it is not yet commercially available (14). Diagnosis is very difficult in case of non pigmented strains. Pathogenecity is not related to pigmentation (15). *C. violaceum* is generally considered to be of low virulence but septicemia can be deadly. The virulent strains of *C. violaceum* have elevated levels of superoxide dismutase and catalase which may protect the bacteria from phagocytic attack in humans, in turn leading to fatal infections (16). Data on antimicrobial susceptibility pattern of this organism is scanty because infections are quite rare. Mostly they are resistant to pencillins and cephalosporins (12). *C. violaceum* infections can be managed with ciprofloxacin, amikacin, gentamicin and meropenem.

## CONCLUSION

*Chromobacterium violaceum* is an uncommon microorganism that can cause fatal sepsis. There are no set guidelines for the management of this infection. *Chromobacterium violaceum* should be considered as a differential diagnosis especially when sepsis is preceded by a skin infection and the susceptibility pattern should be kept in mind while instituting empirical antibiotic therapy. Eventhough many of the published cases in literature are of young adults, our case shows that this organism can cause sepsis even in elderly people.
